# Determination of the presence and antimicrobial resistance of *Arcobacter* species in broiler carcasses at different stages of slaughter line

**DOI:** 10.1002/fsn3.4013

**Published:** 2024-02-06

**Authors:** Yasin Akkemik, Ahmet Güner

**Affiliations:** ^1^ Department of Food Hygiene and Technology Kastamonu University Faculty of Veterinary Medicine Kastamonu Turkey; ^2^ Department of Food Hygiene and Technology Selcuk University Faculty of Veterinary Medicine Konya Turkey

**Keywords:** *Arcobacter* spp., broiler, food safety, public health, slaughterhouse

## Abstract

In this study, to investigate *Arcobacter* spp. contamination post‐scalding and de‐feathering, post‐evisceration, post‐chilling, and packaged products, which are the most essential contamination stages of broiler slaughter, a total of 108 samples were taken from three different broiler slaughterhouses at different times. Isolates obtained by cultural methods in 104 of 108 samples were analyzed by mPCR method to identify pathogen *Arcobacter* spp. *Arcobacter butzleri*, *Arcobacter cryaerophilus*, and mixed contamination of both *Arcobacter* species were detected in 51 samples. Of the 51 isolates, 27 (52.9%) were *A. butzleri*, 16 (31.4%) were *A. cryaerophilus*, and 8 (15.7%) were mixed contamination of *A. butzleri* and *A. cryaerophilus*, while *Arcobacter skirrowii* was not detected. *A. butzleri* and *A. cryaerophilus* contamination was 59.2% post‐scalding and de‐feathering, 43.4% post‐evisceration, 44.4% and 48.1% post‐chilling and in packaged products, respectively. All *A. butzleri* strains were found to be 100% resistant to cefoperazone and penicillin and sensitive to tetracycline. *A. cryaerophilus* strains were 100% resistant to cefoperazone, penicillin, and cloxacillin and susceptible to tetracycline and erythromycin. In the study, it was determined that *Arcobacter* spp. caused a very intense contamination (85.18%–100%) and also contamination rates of identified pathogen strains (*A. butzleri* and *A. cryaerophilus*) were very high (59.2% and 43.4%) in broiler slaughtering stages. Considering that each step in broiler slaughter could contaminate the next stage, developing a safe slaughter and minimizing the risk toward the final product, it was concluded that critical control points could not be well managed in broiler slaughterhouses, and broiler meat may pose a significant risk to public health.

## Graphical abstract

In this study, to investigate *Arcobacter* spp. contamination post‐scalding and de‐feathering, post‐evisceration, post‐chilling, and packaged products. In the study, it was determined that *Arcobacter* spp. caused very intense contamination (85.18%–100%), and also contamination rates of identified pathogen strains (*A. butzleri* and *A. cryaerophilus*) were very high (59.2% and 43.4%) in broiler slaughtering stages.

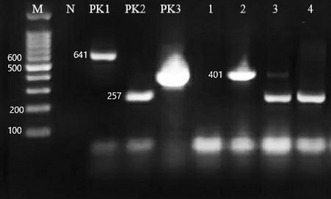

## 1 INTRODUCTION

First isolated from aborted pig fetuses, *Arcobacter* has been initially described as an “aerotolerant *Campylobacter*‐like” bacterium. Because it harbors two aerotolerant species (*A. cryaerophilus* and *Arcobacter nitrofigilis*), it was proposed to be separated from *Campylobacter* in 1991. The most important difference between *Campylobacter* and *Arcobacter* species is the ability of *Arcobacter* to grow at lower temperatures (15–30°C) (Vandamme et al., 1991, 1992; Sciortino et al., 2021).

Biochemical properties of *Arcobacter* species are insufficient to make a definitive identification (Diéguez et al., 2017; Levican et al., 2015; Vandamme et al., 1992). With molecular identification methods, difficulties encountered in the cultural identification of *Arcobacter* spp. have been overcome and the number of species has increased. Up to now, 26 *Arcobacter* species have been identified (Diéguez et al., 2017; Levican et al., 2015). Some species are known to be important enteropathogens for humans and animals (Collado & Figueras, 2011).

The difficulties in diagnosis of the pathogenic *Arcobacter* spp., especially *A. butzleri*, due to its live but nonculturable form (Fera et al., 2008; Zhang et al., 2021), and also its resistance to some antibiotics and its ability to form biofilms (Cervenka, 2007; Ferreira et al., 2013), it is reported to continue to pose a potential hazard to public health. In this context, *A. butzleri* has been accepted as a serious danger to human health and an important zoonotic pathogen by the International Commission on Microbiological Specifications for Foods (ICMSF) (Schönknecht et al., 2020). In a five‐year prevalence study, it has been reported that *Arcobacter* spp. was the fourth most common gastrointestinal pathogen (1.3%) after *Campylobacter* spp. (5.6%), *Salmonella* spp. (2.0%), and *Clostridium difficile* (1.6%) (Nguyen et al., 2022). In a study conducted in Bangkok, *Arcobacter* spp. was reported to have a higher prevalence than other pathogens such as *Salmonella* and *Campylobacter* in raw products (e.g., retail meat, shellfish, vegetables) as well as in restaurant meals (Collado & Figueras, 2011). In a study conducted in Canada, it was reported that *Arcobacter* spp. was found to be 2–3 log higher in irrigation water than *Campylobacter* spp. and, therefore, posed a more significant threat to human health (Banting et al., 2016).

Livestock (e.g., cattle, poultry, pigs), wastewater, and aquaculture (e.g., fish, shellfish) are the main reservoirs for *Arcobacter* spp., which are widespread in various habitats (Collado & Figueras, 2011; Hsu & Lee, 2015; Mudadu et al., 2021). Contamination by *A. butzleri*, *A. skirrowii*, *A. creoaerophilus* has also been reported in dairy products such as milk and cheese and in vegetables (e.g. spinach, lettuce) (Ferreira et al., 2016, 2017; Lehner et al., 2005). However, animal‐derived foods, especially poultry and pork, have been reported to have a high prevalence of *Arcobacter* spp. (Collado & Figueras, 2011; Hsu & Lee, 2015).

The acquisition of antibiotic resistance by bacteria through different mechanisms and contamination of foods with these resistant bacteria may pose severe problems for public health. Especially in developing countries, the prevalence of antibiotic resistance among foodborne pathogens is gradually increasing due to the irregular use of antibiotics (Jribi et al., 2020). In a meta‐analysis study, resistance rates were between 69.3%–99.2% for penicillins and 30.5%–97.4% for cephalosporins. The same survey reported that the resistance rate to fluoroquinolones was between 4.3% and 14.0%, and the highest resistance rate was found against levofloxacin. However, resistance rates ranged between 10.7%–39.8% for macrolides, 1.8%–12.9% for aminoglycosides, and 0.8%–7.1% for tetracyclines (Ferreira et al., 2019).

*Arcobacter* spp. like *Campylobacter* spp. are known as foodborne microorganisms as they are isolated from broiler meat worldwide (Ferreira et al., 2019; Son et al., 2007). In studies conducted on the same broiler carcasses, it was reported that the isolation rates of *Arcobacter* spp. were higher than those of *Campylobacter* spp. in almost all samples (Houf et al., 2002b). *Arcobacter* spp. has been isolated from different stages of the broiler slaughter line (e.g., scalding and de‐feathering, evisceration, pre‐ and post‐chilling), equipment, and processing water (Ho et al., 2008). It was reported that *Arcobacter* spp. could not be isolated from the intestinal contents of broilers belonging to the same flock from which the carcasses were came (Atabay & Corry, 1997). This indicates that the source of *Arcobacter* contamination in carcasses may be the slaughterhouse environment and/or the water used during processing (Atabay & Corry, 1997; Ho et al., 2008). Three of the four reported *Arcobacter* outbreaks have been linked to the consumption of water contaminated with feces, and the other to the consumption of fried chicken (Collado & Figueras, 2011; Ferreira et al., 2016). An incidence ranging from 13.6% (Denmark) (Hsu & Lee, 2015) to 84.6% (Belgium) (Houf et al., 2002a) has been determined in poultry processing plants, depending on the country.

In this study, it was aimed to determine the presence and contamination sources of *Arcobacter* spp. and three pathogenic species (*A. butzleri*, *A. skirrowii*, *A. creoaerophilus*) at different stages of broiler slaughter line and to determine their antimicrobial resistance.

## 2 MATERIALS AND METHODS

Three broiler slaughterhouses (approved by the government according to Regulation No. 28145 on the Official Control of Animal Food) were visited thrice. During each visit, 36 samples taken from four different stages of slaughter (post‐scalding and de‐feathering, post‐evisceration, post‐chilling, packaged product) were placed in sterile bags as whole carcasses and brought to the laboratory in a cold chain. At the end of the three visits, totally 108 samples were taken. Isolation and identification of *Arcobacter* spp. from a total of 108 samples were carried out using the cultural method; for confirmation and species‐level identification of the isolates obtained, multiplex polymerase chain reaction (mPCR) was performed. The antibiogram test was used to determine antibiotic susceptibilities.

Broiler carcasses taken post‐scalding and de‐feathering were washed with a sponge in sterile bags using 400 mL Maximum Recovery Diluent (MRD‐Merck‐SK.M112535.0500‐Germany) solution. The samples collected from the other stages were placed in sterile bags, 400 mL of MRD was added, and the internal and external surfaces of the carcass were thoroughly washed by shaking (approximately 35 rpm) for 1 min (Capita et al., 2004; Benli et al., 2015). Ten milliliter of the washing water was transferred to sterile Pyrex bottles.

The method proposed by Lehmann et al. (2015) was used to isolate and identify *Arcobacter* spp. 90 mL *Arcobacter* selective broth (ASB‐HIMEDIA‐M1894‐India) with cefoperazone, amphotericin B, and teicoplanin (CAT Selective Supplement‐HIMEDIA‐FD145‐India) was added to 10 mL rinse water. The broth was incubated at 30°C for 48 h under microaerobic conditions. After incubation, the broth was diluted 1/10 with Brucella Broth (BB‐HIMEDIA‐M348‐India) and 0.3 mL was spread on the filter (Filter‐Lab‐MCE045047WGSN) on 5% sheep blood Mueller Hinton Agar (MHA‐Merck‐SK.M105437.0500‐Germany) and incubated under aerobic conditions for 1 h, and the filter was removed with a sterile forceps. 0.1 mL BB was added to MHA and incubated aerobically at 30°C for 48 h. Suspicious colonies were switched to MHA and incubated at 30°C for 48 h under microaerobic conditions. After incubation, Gram staining, oxidase, catalase, nitrate reduction, urease, and hippurate tests were performed. In addition, isolates of *Arcobacter* spp. were transferred to a BB storage medium containing 15% glycerin and stored at −86°C for molecular identification and antibiotic susceptibility tests.

Isolates and reference strains (*A. butzleri*‐DSM8739, *A. cryaerophilus*‐DSM7289, *A. skirrowi*‐DSM 7302) in glycerin BB storage medium were transferred to centrifuge tubes containing Nutrient Broth (HIMEDIA‐M439SIndia). After incubation at 37°C, the isolates were suspended in centrifuge tubes containing 50 μL of sterile bidistilled water with the help of a pipette. Centrifuge tubes were kept in a heat block (Bioneer MyGenie 96 Thermal Block‐Cycler) at 110°C for 15 min and then centrifuged (Nüve NF 1200 R) at 1200 *g* value for 15 min. The DNA supernatants obtained were placed in sterile centrifuge tubes and used in mPCR analysis.

PCR Master Mix (2X) (Thermo Fisher‐K0171) was used in mPCR analysis for molecular identification of *Arcobacter* spp. isolates. The mixture was prepared in sterile centrifuge tubes using 10 μL PCR master mix, 1 μL each of ARCO, BUTZ, CRY1, and CRY2, and 0.5 μL SKIR primers (Sentebiolab), 2 μL isolate DNA, and 12.55 μL sterile bidistilled water to a total volume of 25 μL. PCR amplification was performed at 95°C for 5 min (initial denaturation), 94°C for 1 min (denaturation), 55°C for 1 min annealing, 72°C for 2 min (primer extension), and 72°C for 15 min (final extension) for 40 cycles. The amplified PCR products were detected by electrophoresis in 1.5% agarose gel at 90 V, 70 mA, and 60 min.

The resistance pattern of the isolates was determined by disk diffusion (Hudzicki, 2009) for cefoperazone (Bioanalyse‐CEP200262, 75 μg‐Turkey), tetracycline (Bioanalyse‐TE201429, 30 μg‐Turkey), ampicillin (Bioanalyse‐AM200026, 10 μg‐Turkey), erythromycin (Bioanalyse‐E200590, 15 μg‐Turkey), cloxacillin (Bioanalyse‐ST00021, 1 μg‐Turkey), and penicillin (Bioanalyse‐P201153, 10 IU‐Turkey). Since there is no standard zone range for *Arcobacter* spp., the zones formed on blood agar (BA‐Merck‐1.10886‐Germany) containing 5% sheep blood were evaluated according to the criteria determined by the Clinical and Laboratory Standards Institute (CLSI, 2020).

Statistical analyses of results obtained in the study were performed by Pearson chi‐square and Fisher's exact chi‐square tests using the SPSS package program (SPSS Version 21).

## 3 RESULTS

In the study, to determine the contamination levels of *Arcobacter* spp. post‐scalding and de‐feathering, post‐evisceration, post‐chilling, and packaged products, which have the highest contamination risk in broiler slaughtering processes, isolation, and identification procedures were carried out by cultural and molecular methods in 108 samples obtained from various slaughterhouses at different times.

### 3.1 Cultural isolation and identification

**FIGURE 1:**
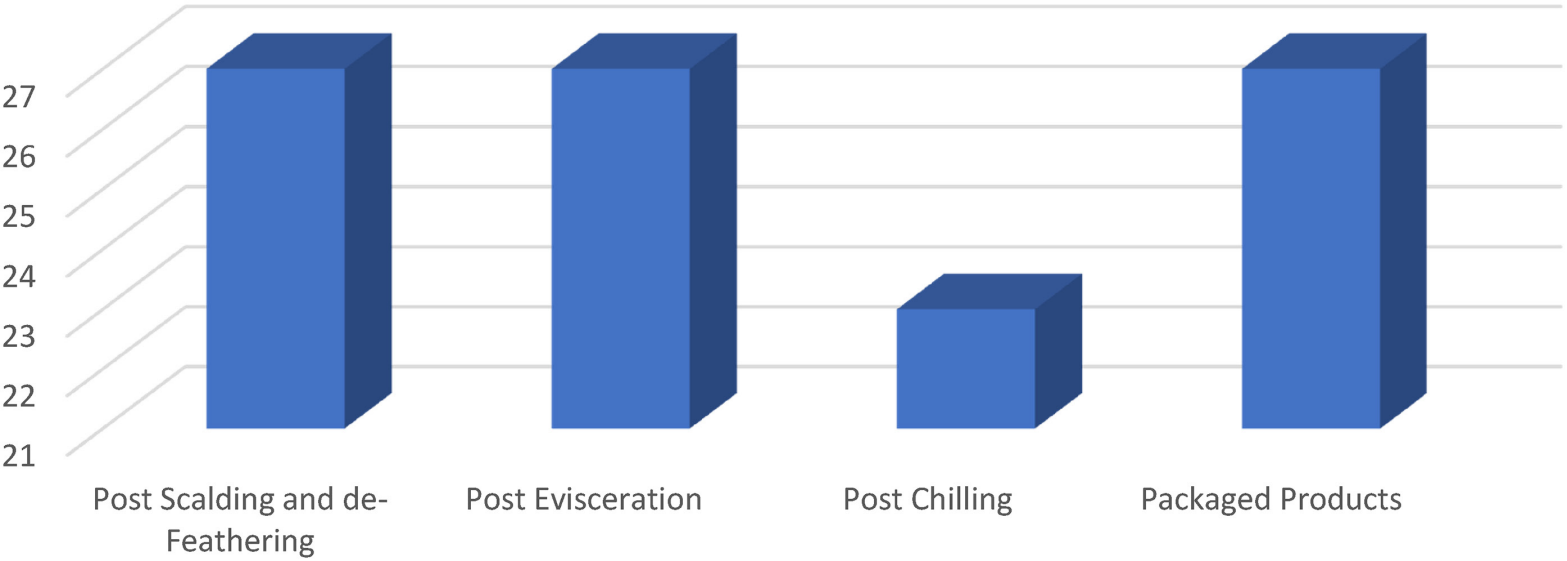
Results of cultural isolation.

After cultural isolation and identification, suspicious colonies of *Arcobacter* spp. were detected in 104 of 108 samples. The distribution of suspicious colonies according to the slaughter stages is given in Figure 1.

### 3.2 Molecular identification

**FIGURE 2:**
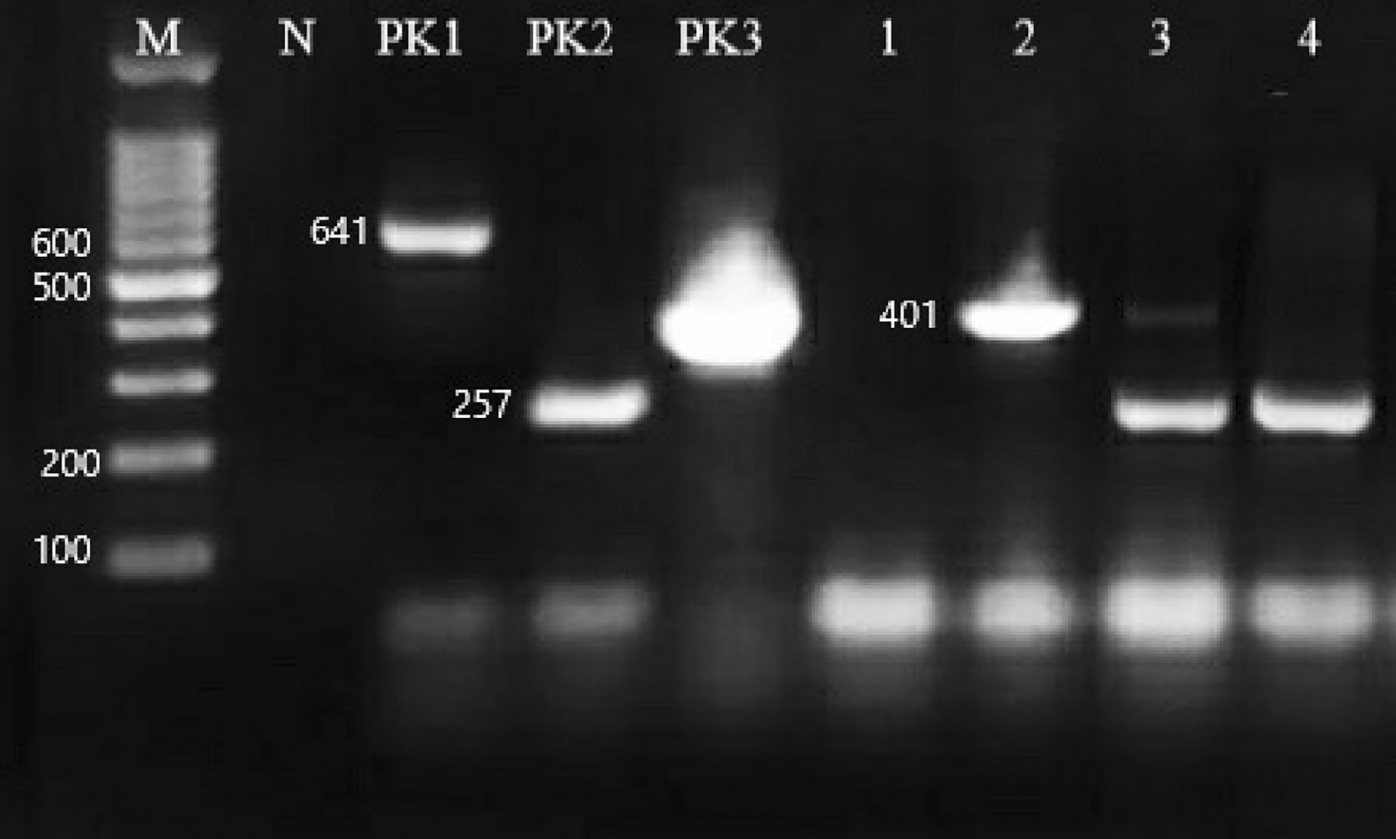
Results of molecular identification. M, marker (100 bp DNA Ladder 50μg Hibrigen MG‐LDR‐100‐1 1‐Turkey); N, negative control; PK1, *A. skirrowii* positive control; PK2, *A. cryaerophilus* positive control; PK3, *A. butzleri* positive control; 1, negative sample; 2, *A. butzleri* positive sample; 3, *A. cryaerophilus* and *A. butzleri* positive mixed contamination sample; 4, *A. cryaerophilus* positive sample.

After mPCR and electrophoresis applied to the reference strains and the isolates obtained as *Arcobacter* spp. by cultural methods, *A. butzleri* was observed at 401 bp, *A. cryaerophilus* at 257 bp, and *A. skirrowii* at 641 bp (Figure 2).

After mPCR was applied to 104 isolates identified as *Arcobacter* spp. by cultural method 51 (49.03%) of the isolates were identified as *Arcobacter* spp. When the species‐based distribution was analyzed, it was found that *A. butzleri* was more common (52.9%), followed by *A. cryaerophilus* (31.4%) and *A. butzleri*–*A. cryaerophilus* (15.7%) mixed contamination.

Statistical analysis on the distribution of pathogen *Arcobacter* spp. contamination detected at different slaughtering stages showed no correlation between pathogen *Arcobacter* spp. (*p* > .05) (Table 1).

**TABLE 1:** Distribution of pathogen *Arcobacter* species contamination at slaughter stages.

	*Arcobacter* spp.	Positive (*n*)	%
Post‐scalding and de‐feathering	*A. Butzleri*	7	43.8
	*A. Cryaerophilus*	6	37.5
	*A. Butzleri* + *A. Cryaerophilus*	3	18.8
	Total	16	100.0
Post‐evisceration	*A. butzleri*	6	60.0
	*A. cryaerophilus*	3	30.0
	*A. butzleri* + *A. cryaerophilus*	1	10.0
	Total	10	100.0
Post‐chilling	*A. butzleri*	6	50.0
	*A. cryaerophilus*	5	41.7
	*A. butzleri* + *A. cryaerophilus*	1	8.3
	Total	12	100.0
Packaged products	*A. butzleri*	8	61.5
	*A. cryaerophilus*	2	15.4
	*A. butzleri* + *A. cryaerophilus*	3	23.1
	Total	13	100.0
	Chi‐square value	3.381	
	*p*‐value (*p* > .05)	.760	

### 3.3 Antibiotic susceptibility test results

The six antibiotic types (cefoperazone, tetracycline, ampicillin, erythromycin, penicillin, and cloxacillin) used in the study were selected by considering the antibiotic sensitivities against *Arcobacter* species detected in different foods (Ferreira et al., 2019) and in poultry facilities (Jribi et al., 2020; Rahimi, 2014). All *A. butzleri* isolates were 100% resistant to cefoperazone and penicillin, 96.3% to cloxacillin, 66.6% to ampicillin, and 3.7% to erythromycin. However, all of the *A. butzleri* isolates were susceptible to tetracycline.

*A. cryaerophilus* isolates were 100% resistant to cefoperazone, penicillin, and cloxacillin, 87.5% resistant to ampicillin, and 100% susceptible to tetracycline and erythromycin.

**FIGURE 3:**
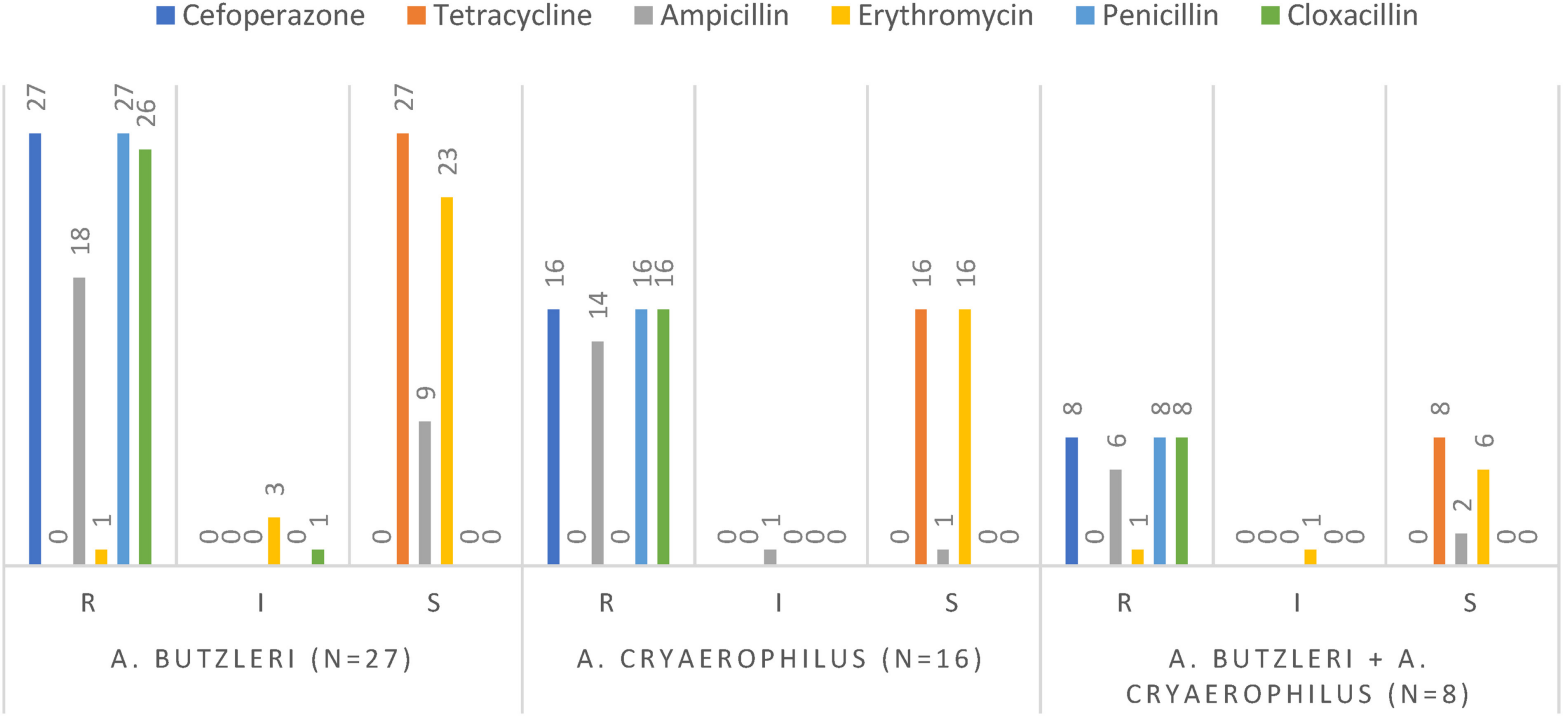
Results of antibiotic susceptibility test. I, intermediate; R, resistant; S, susceptible.

*A. butzleri* and *A. cryaerophilus* mixed isolates were found to be 100% resistant to cefoperazone, penicillin, and cloxacillin, 75% resistant to ampicillin, and 12.5% resistant to erythromycin, while all mixed isolates were susceptible to tetracycline (Figure 3).

## 4 DISCUSSION

In the study, it was investigated the presence of *Arcobacter* spp. in broiler meat, the most consumed meat type in Turkey, at the slaughtering stages. *Arcobacter* spp. was isolated from 104 of 108 samples (96.29%). Fifty‐one (49.03%) of the 108 samples were contaminated with pathogenic *Arcobacter* spp. Of the pathogenic isolates, 27 (52.9%) were contaminated with *A. butzleri*, 16 (31.4%) with *A. cryaerophilus*, and 8 (15.7%) with *A. butzleri* and *A. cryaerophilus*.

*A. butzleri* and *A. cryaerophilus* contamination rates post‐scalding and de‐feathering, post‐evisceration, post‐chilling, and packaged products were 59.2%, 43.4%, 44.4%, and 48.1%, respectively. In 51 samples, of the two pathogenic species, *A. butzleri* was detected at a higher rate (52.9%). It is thought that this may be due to the suppression of the growth of other species in enrichment cultures due to the rapid growth of *A. butzleri* (Adesiji et al., 2014; Sasi Jyothsna et al., 2013).

The study's uniqueness is that there are a limited number of studies examining different stages of broiler slaughter line in terms of *Arcobacter* spp., and this is the first study conducted in Turkey. Son et al. (2007) determined the contamination levels with *Arcobacter* spp. as 96.8% before scalding, 61.3% before chilling, and 9.6% post‐chilling. Similar to the findings of this study, the same researchers (Son et al., 2007) reported that *A. butzleri* was the most common species (79.1%), followed by *A. cryaerophilus* 1B (18.6%) and *A. cryaerophilus* 1A (2.3%) among *Arcobacter* spp. obtained at different stages of broiler slaughtering.

Atanassova et al. (2008) determined the contamination levels with *Arcobacter* spp. as 48% before and post‐evisceration and 60% post‐chilling. They reported that the presence of *Arcobacter* spp. in broiler slaughtering stages increased post‐chilling. The increase determined by the researchers in the samples taken from the cooling stage applied post‐evisceration is in parallel with the findings obtained in this study. In addition, similarly to our study, the researchers reported that *A. butzleri* was the most common species among *Arcobacter* spp. at different stages of broiler slaughtering, followed by *A. skirrowii* and *A. cryaerophilus*, respectively.

Khoshbakht et al. (2014) determined the contamination levels with *Arcobacter* spp. as 30% before scalding, 48% post‐scalding, 73% post‐evisceration, and 18% at the 24th hour of cooling. The researchers' findings post‐scalding and evisceration are similar to the values obtained in this study. It is thought that the difference before scalding and post‐chilling may be due to the effectiveness of decontamination methods and the differences between the enterprises. In the same study, similar to the findings of this research, it was determined that the most common species among *Arcobacter* spp. in different stages of broiler slaughtering was *A. butzleri*, with 31.25%.

Similar to the findings of this study, Ho et al. (2008) detected *Arcobacter* spp. in 95 of 100 broiler carcasses collected from different stages of broiler slaughtering. As a result of mPCR applied to 95 isolates, 58 samples (61.1%) were *A. butzleri*, 1 sample (1.1%) was *A. cryaerophilus*, 29 samples (30.5%) were *A. butzleri* and *A. cryaerophilus* mixed contamination, 7 samples (7.36%) were *A. butzleri* and *A. skirrowii* mixed contamination. Atabay et al. (2006) isolated and identified *Arcobacter* spp. in 85 (85%) of 100 samples (30 whole carcasses, 70 cloacal swabs, and feces) collected from broiler slaughterhouses. Houf et al. (2002b) investigated the evisceration and post‐chilling stages of 8 different broiler slaughterhouses. They found *Arcobacter* spp. contamination in 919 of 960 samples (95.7%).

In a study carried out by Khoshbakht et al. (2014), in which *A. butzleri* was the most frequently detected species and the distribution was similar to the findings obtained at the slaughtering stages in this study, *A. butzleri* was 75%, *A. cryaerophilus* 2%, *A. skirrowi* 0% and mixed contamination of *A. butzleri* and *A. cryaerophilus* was 17% before scalding. post‐scalding; *A. butzleri* 79%, *A. cryaerophilus* 0%, *A. skirrowi* 11%, and mixed contamination of *A. butzleri* and *A. cryaerophilus* 11%, post‐evisceration; *A. butzleri* 69%, *A. cryaerophilus* 14%, *A. skirrowi* 3.4% and *A. butzleri* and *A. cryaerophilus* mixed contamination was 14%, and *A. butzleri* 86%, *A. cryaerophilus* 14%, *A. skirrowi* 0% and *A. butzleri* and *A. cryaerophilus* mixed contamination 0% at 24 hours of cooling.

However, Harrass et al. (1998) detected *Arcobacter* spp. at a lower rate compared to the findings obtained in our study. The researchers detected *Arcobacter* spp. in 89 (52.35%) of the samples obtained from the skin and intestinal contents of 170 freshly slaughtered broilers and reported that all positive samples were of skin origin. The reason for this difference is thought to be that the researchers took the samples only from the skin and intestinal contents. Rahimi (2014) also detected *Arcobacter* spp. at a lower rate compared to the findings obtained in our study. The researcher reported detecting 28 (28%) *Arcobacter* spp. with cultural techniques applied to 100 broiler carcasses. This difference is because the researcher took samples only from packaged products, which are the final products. However, Son et al. (2007) detected 12 (*n* = 125) *Arcobacter* spp. in carcasses obtained post‐chilling. However, it is seen that higher rates were detected at different stages of slaughter. Lee et al. (2010) identified 38 samples (10.6%) as *A. butzleri* according to the results of oxidase, catalase, nitrate reduction, and 2,3,4‐triphenyltetrazolium chloride resistance tests performed on suspicious colonies obtained in the medium as a result of cultural isolation and identification of 360 broiler samples, while *A. cryaerophilus* and *A. skirrowii* were not detected.

Results obtained in the disk diffusion method applied to determine the antibiotic resistance of the pathogen *Arbobacter* ssp. isolates identified in the study, Atabay and Aydin (2001) found similar resistance findings to our study. They determined 100% resistance to aztreonam, cefuroxime sodium, cephalothin, orbenin, oxacillin, penicillin G, and trimethoprim/sulfamethoxazole, amoxicillin, amoxicillin/clavulanic acid and ampicillin in 26 of 39 *A. butzleri* strains isolated from broiler chickens. One isolate was resistant to erythromycin, and 4 isolates were moderately resistant to erythromycin, and all isolates were susceptible to amikacin, chloramphenicol, danofloxacin, enrofloxacin, nitrofurantoin, nalidixic acid, tetracyclines, and tobramycin. Similar to the findings of this study, Rahimi (2014) reported that 64 *A. butzleri*, *5 A. cryaerophilus*, and 2 *A. skirrowii* isolated from poultry meat (chicken, turkey, quail, partridge, duck, ostrich) were all susceptible to tetracycline. In the same study, similar to our study, 1.6% and 0% resistance to erythromycin were found, respectively.

## 5 CONCLUSION

Although *Arcobacter* spp. is less known than other foodborne pathogens and there is no legal regulation regarding its presence in foods (Collado & Figueras, 2011), it has been reported that the isolation amounts of *Arcobacter* spp. are higher than *Campylobacter* spp. in studies conducted on broiler carcasses. In this study, as in many previous studies, 100% of the samples examined post‐scalding and de‐feathering, post‐chilling, and packaged products were contaminated with *Arcobacter* spp., 85.15% of the samples examined post‐evisceration, and 49.03% of the contaminated samples carried pathogenic *Arcobacter* spp. According to these results, it was concluded that the source of *Arcobacter* contamination in broiler carcasses may be the slaughterhouse environment and/or the water used during processing and that it is essential to pay utmost attention to hygiene rules inside and outside the slaughterhouse. Considering the application of HACCP rules in broiler slaughtering, each stage should be developed by considering the next step, and the risk should be minimized toward the final product. The findings obtained in the research concluded that HACCP could not be well managed in broiler slaughterhouses, and broiler meat may pose a significant risk to public health. It is essential to comply with the basic principles of HACCP and good production practices in slaughterhouses and to raise awareness by providing periodic training to the personnel. Considering the increase in the production, consumption, import, and export of broiler meat in the world and Turkey, it is thought that it is essential to carry out more studies on the subject and implement protective measures to consume healthier broiler meat.

## AUTHOR CONTRIBUTIONS

**Yasin Akkemik:** Data curation (equal); formal analysis (equal); methodology (equal); resources (equal); writing – original draft (equal); writing – review and editing (equal). **Ahmet Güner:** Data curation (equal); formal analysis (equal); methodology (equal); resources (equal); writing – original draft (equal); writing – review and editing (equal).

## FUNDING INFORMATION

This research was supported by Selçuk University Scientific Research Projects Coordination Office with 21112001 project code and PhD Thesis project type.

## CONFLICT OF INTEREST STATEMENT

The authors declare that they do not have any conflicts of interest.

## ETHICS STATEMENT

This study was approved by the local ethics committee of the Faculty of Veterinary Medicine, which works under the Central Ethics Committee of Turkey.

## Data Availability

Data available on request due to confidentiality/ethical restrictions.
